# Factors associated with hip deformity in children with nonambulatory spastic cerebral palsy

**DOI:** 10.1097/MD.0000000000049553

**Published:** 2026-06-26

**Authors:** Ju Seok Ryu, Yulhyun Park, Joonyoung Jang, Hyun Jin Kim, Eunseo Choi, Jaewon Lee, Jee Hyun Suh

**Affiliations:** a Department of Rehabilitation Medicine, Seoul National University Bundang Hospital, Seoul National University College of Medicine, Seongnam, South Korea; b Department of Rehabilitation Medicine, Icheon Medical Center, Icheon, Republic of Korea; c Department of Rehabilitation Medicine, Korea Workers’ Compensation & Welfare Service Daegu Hospital, Daegu, Republic of Korea.

**Keywords:** cerebral palsy, coxa valga, hip deformity, migration index, surface electromyography

## Abstract

Hip deformities, including subluxation, dislocation, and coxa valga, are common in nonambulatory children with spastic cerebral palsy (CP), especially those classified as Gross Motor Function Classification System IV and V. These deformities impact daily activities and quality of life. This study aimed to identify factors associated with hip deformity and explore rehabilitation strategies to prevent complications. A cross-sectional study (August 2018–May 2020) included 46 children with spastic CP (Gross Motor Function Classification System IV-V, aged 2–10 years). Radiographic and physical measurements, including range of motion, spasticity, muscle strength, and surface electromyography, were analyzed using multiple regression and Pearson correlation. Hip deformities were assessed via Reimer’s migration index, femoral neck-shaft angle, acetabular index, and pelvic obliquity. Migration index was negatively correlated with hip abduction range of motion (ROM) at 90-degree flexion. The femoral neck-shaft angle showed a negative correlation with the abductor-to-adductor muscle strength ratio. These findings suggest that reduced hip abduction ROM and muscle strength imbalance are associated with hip deformities in nonambulatory children with spastic CP. Hip abduction ROM and muscle strength balance are crucial for preventing hip deformities in nonambulatory children with spastic CP. While strengthening hip abductors and improving ROM may be potentially beneficial, interventional studies are needed to confirm whether these strategies reduce subluxation risk. Early identification using noninvasive methods like ROM and surface electromyography can help minimize radiation exposure from frequent radiographic evaluations.

## 1. Introduction

Cerebral palsy (CP) is a group of permanent disorders of the development of movement and posture, causing activity limitation, that are attributed to nonprogressive disturbances that occurred in the developing fetal or infant brain.^[1]^ The motor disorders of cerebral palsy are often accompanied by disturbances of sensation, perception, cognition, communication, and behavior, by epilepsy, and by secondary musculoskeletal problems. CP is generally categorized into 4 types: spastic, dyskinetic, ataxic, and mixed. Among them, spastic CP is the most prevalent, making up approximately 70% to 80% of all cases.^[2]^ Although neurological impairment remains stable, musculoskeletal complications tend to worsen over time in children with CP. Among the various musculoskeletal issues associated with CP, hip deformities are particularly prevalent, ranking second only to foot and ankle abnormalities.^[3,4]^ These hip deformities encompass a wide range of conditions, including hip subluxation and dislocation, as well as coxa valga, acetabular deformities, and increased pelvic obliquity (PO). These conditions affect nearly one-third of children with CP, with a higher occurrence in nonambulatory children with CP classified as Gross Motor Function Classification System (GMFCS) IV and V, ranging from 3.9% per year at GMFCS level IV to 9.5% at level V.^[5,6]^ A recent systematic review and pooled analysis confirmed that the cumulative incidence of hip displacement reaches 71.9% in nonambulatory children with CP (GMFCS IV-V), underscoring the severity of this problem in this population.^[7]^ Hip deformities in CP range from early-stage concerns, such as at-risk hips, to more severe conditions, including subluxation, dislocation, and degenerative arthritis accompanied by pain. If left unmanaged, the progression of hip disorders can result in significant discomfort and challenges related to sitting and maintaining proper hygiene.^[8]^ Furthermore, a scoping review by Malone et al demonstrated that progressive hip displacement adversely impacts functional outcomes across multiple International Classification of Functioning, Disability and Health (ICF) domains in children and adolescents with CP, highlighting the importance of early identification and intervention.^[9]^

Our previous study suggested that rehabilitation treatment goals should be tailored based on ambulatory status.^[10]^ For ambulatory individuals, the primary focus should be on functional improvement, whereas for nonambulatory individuals, the priority should be preventing complications, such as hip subluxation and scoliosis. Therefore, in patients with nonambulatory CP, it is essential to establish evaluation metrics that are distinct from those used for ambulatory CP, incorporating indicators such as the hip migration index (MI) or Cobb’s angle for scoliosis progression. Notably, a recent study demonstrated that femoral neck-shaft angle increases progressively with worsening neurological impairment across GMFCS levels, and that the role of spasticity in the development of coxa valga remains an area of active investigation, reinforcing the need for objective biomechanical assessment in this population.^[11]^

This study aimed to identify the factors associated with hip deformity in nonambulatory children with spastic CP and to discuss potential rehabilitation implications based on the observed associations between hip deformity and physical measurements, including the range of motion (ROM) and muscle activation patterns. This study hypothesized that ROM of certain joints or the activation of specific muscles may be linked to various hip deformities in nonambulatory children with spastic CP.

## 2. Methods

### 2.1. Participants

This study was conducted and reported in accordance with the Strengthening the Reporting of Observational Studies in Epidemiology guidelines for cross-sectional studies.^[12]^ This cross-sectional study was conducted in 3 rehabilitation units in the Republic of Korea between August 2018 and May 2020. Patients aged 2 to 10 years and diagnosed with spastic CP and GMFCS levels IV-V were included. The exclusion criteria were as follows: neuromuscular disorders such as muscular dystrophy or poliomyelitis; lack of consent for study participation; and children with CP that were not of the spastic type.

Medical information regarding sex, age, and surgical history was also collected. Functional levels were assessed by physiatrists using the GMFCS.^[13]^ Written informed consent was obtained from the parents or legal guardians of all participating children with CP, and the study protocol was approved by the institutional review board (B-1808-484-302). This study was conducted in accordance with the ethical principles of the Declaration of Helsinki.

### 2.2. Radiographic measurements

**Figure 1.**
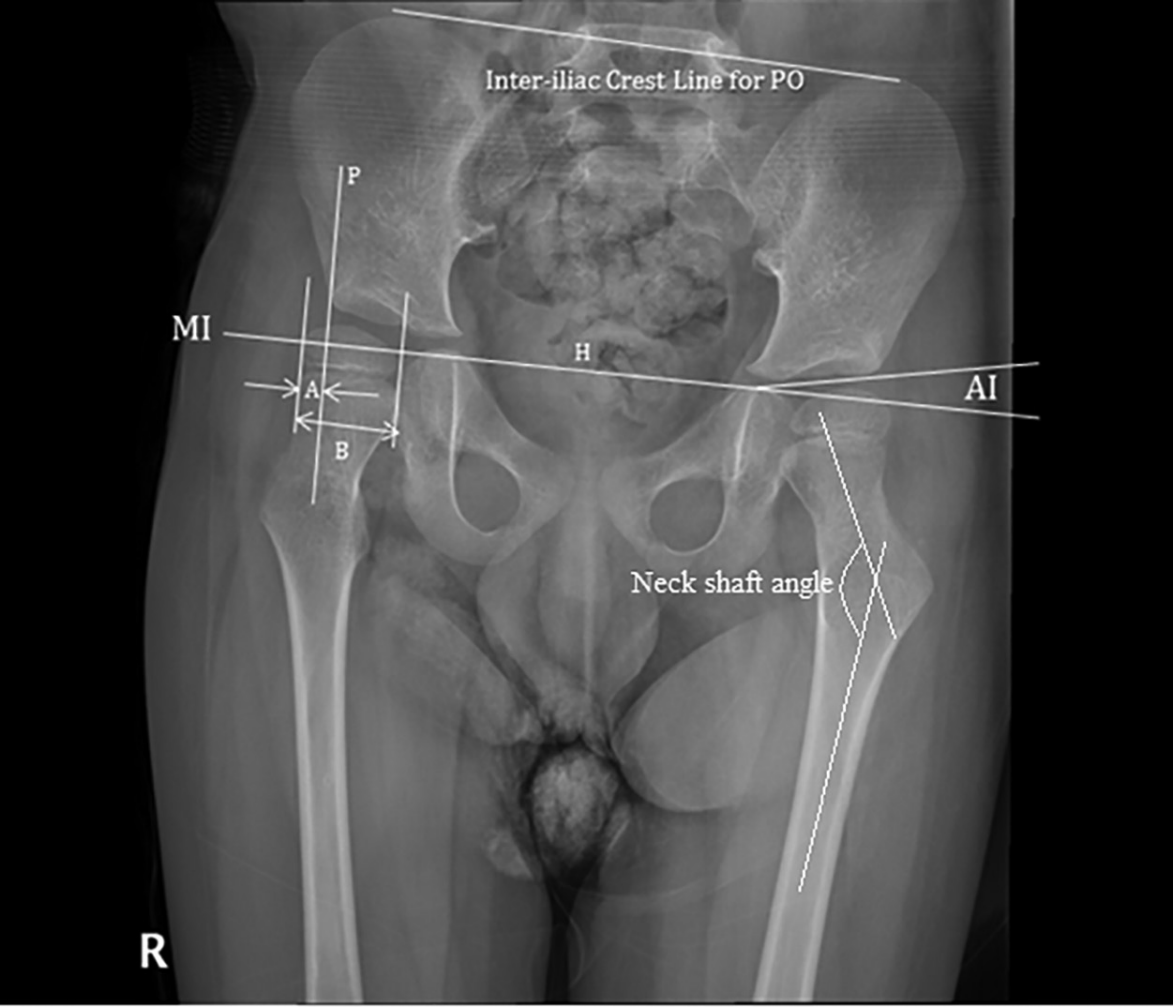
Hip radiography method for measuring MI, AI, neck-shaft angle, and PO.

Plain radiographic images of the pelvis and hip joints in the anteroposterior direction of the patients in the supine position were obtained. Radiographic parameters included Reimer’s MI, femoral neck-shaft angle, acetabular index (AI), and PO. Reimer’s MI is the percentage of the ossified femoral head positioned lateral to the acetabular margin on anteroposterior pelvic radiography.^[14]^ The femoral neck-shaft angle was measured to determine the coxa valgus of the hip. The angle was made up of 2 lines: one connecting the center of the femoral neck to the head and another drawn through the femoral shaft midline.^[15]^ AI was measured as the angle between the slope of the acetabular roof and Hilgenreiner’s line when the triradiate cartilage was open.^[16]^ The PO was measured as the angle between the horizontal reference line parallel to the frame of the radiograph, with the line joining either the acetabular teardrops or a line joining the maximum prominence of the iliac crest^[17]^ (Fig. 1).

### 2.3. Physical measurements: range of motion (ROM), spasticity, strength of the hip adductor muscles

The ROM of hip abduction with 90-degree hip flexion and hip extension, hip internal rotation, hip external rotation, knee flexion, and knee extension was measured by a physiatrist using a goniometer.

Spasticity was measured using the Modified Ashworth scale (MAS).^[18]^ In this study, MAS was used to assess the degree of muscle spasticity in the hip adductor with knee extension, knee flexion, hip flexion, and knee extension.

Hip adductor muscle strength was measured using a manual muscle tester (Model 01165, Lafayette Manual Muscle Tester; Fabrication Enterprises Inc.). To measure the force exerted between both legs during spasticity, children with CP were seated in a chair with a gap of approximately 10 cm between their distal femurs. A manual muscle tester was placed at the midpoint of the femur, and the force applied to the device during spasticity was recorded.

### 2.4. Surface electromyography (S-EMG)

To evaluate the degree of muscular contraction of the hip abductors and adductors and the degree of activation of core muscles, a wireless surface electromyography (S-EMG) analysis system (BTS FREEEMG 1000 with EMG-BTS EMG-Analyzer, BTXD Bioengineering Co) was used for electrophysiological quantitative analysis. S-EMG electrodes were attached to the belly of the major adductor muscles (adductor longus muscle, adductor magnus muscle), abductor muscles (tensor fascia lata muscle, gluteus medius muscle), and core muscles (rectus abdominis, external abdominal oblique, L3 erector spinae, and L3 multifidus) bilaterally.^[6,19,20]^ The anatomical criteria of the attachment site of the S-EMG electrodes are shown in Supplemental Table 1, Supplemental Digital Content 1. All electrodes were applied by the same trained physiotherapist following standardized anatomical landmarks, and skin preparation, including cleansing with alcohol swabs, was performed prior to electrode application to minimize impedance and ensure signal quality.

S-EMG examination was performed as follows: when the patients rested and when spasticity occurred. Recordings with excessive noise or movement artifacts were excluded and repeated. S-EMG was performed twice, and the mean value (in microvolts) was calculated. The root mean square (RMS) was measured for 1 second on the plateau.

The adductor sum refers to the total mean RMS amplitude (microvolts) measured from the 2 primary adductor muscles, whereas the abductor sum is the total mean RMS amplitude (microvolts) measured from the 2 main abductor muscles using S-EMG data.^[21]^ The Net Adduction Index is calculated by summing the products of the moment arm length and mean RMS amplitude of the 2 primary adductors and subtracting the sum of the products of the moment arm length and mean RMS amplitude of the 2 main abductors. This index represents the weighted sum of the strengths of the adductor and abductor muscles, with weighting based on the moment-arm length of each muscle. The moment-arm lengths for the adductor longus and adductor magnus (anterior head) were 7.1 cm and 6.9 cm, respectively, whereas the moment-arm lengths for the gluteus medius (middle fibre) and tensor fascia lata were 6.0 cm and 5.2 cm, respectively.^[21]^ The net adduction index is the most accurate indicator of the adduction force applied to the femoral head. The formula for calculating the net adduction index was as follows:


$$\begin{array}{c} \begin{array}{cc} & \Sigma \;mean\;f\;RMS\;of\;adductor\;m.\;amplitude\;(\mu V)\\ & \times \;momentum\text{-}arm\;length\;(cm)\\ & -\Sigma \{mean\;RMS\;of\;abductor\;m.\;amplitude\;(\mu V)\\ & \times \;momentum\;arm\text{-}length\;(cm)\} \end{array} \end{array}$$

### 2.5. Statistical analysis

All statistical analyses were conducted using SPSS 22.0 software (SPSS Inc., Chicago, IL). A *P*-value of <.05 was considered statistically significant.

Descriptive statistics were reported as mean ± standard deviation (SD) for continuous variables and as frequency with percentage for categorical variables.

The normality of continuous variables was assessed using the Shapiro–Wilk test, which is appropriate for small sample sizes (*n* = 46). Variables that satisfied the normality assumption were analyzed using parametric methods.

Pearson’s correlation analysis was conducted to examine the bivariate relationships between hip deformity parameters (MI, femoral neck-shaft angle, AI, and PO) and physical measurements, including ROM of the lower extremities, hip adductor muscle strength, and S-EMG variables (adductor sum, abductor sum, and net adduction index). Pearson’s correlation was selected given the continuous nature of all variables and the satisfaction of normality assumptions. The correlation coefficient (*r*) was interpreted according to the criteria of Dancey and Reidy: *R* = 0.1–0.3 as weak, *R* = 0.3–0.6 as moderate, and *R* > 0.6 as strong.^[22]^

Additionally, Pearson’s correlation analysis was conducted to examine the relationships between MI, femoral neck-shaft angle, AI, PO, adductor sum, abductor sum, net adduction index, ROM of the lower extremities, strength of the hip adductor, and S-EMG results.

## 3. Results

### 3.1. Clinical characteristics of the participants

**Figure 2.**
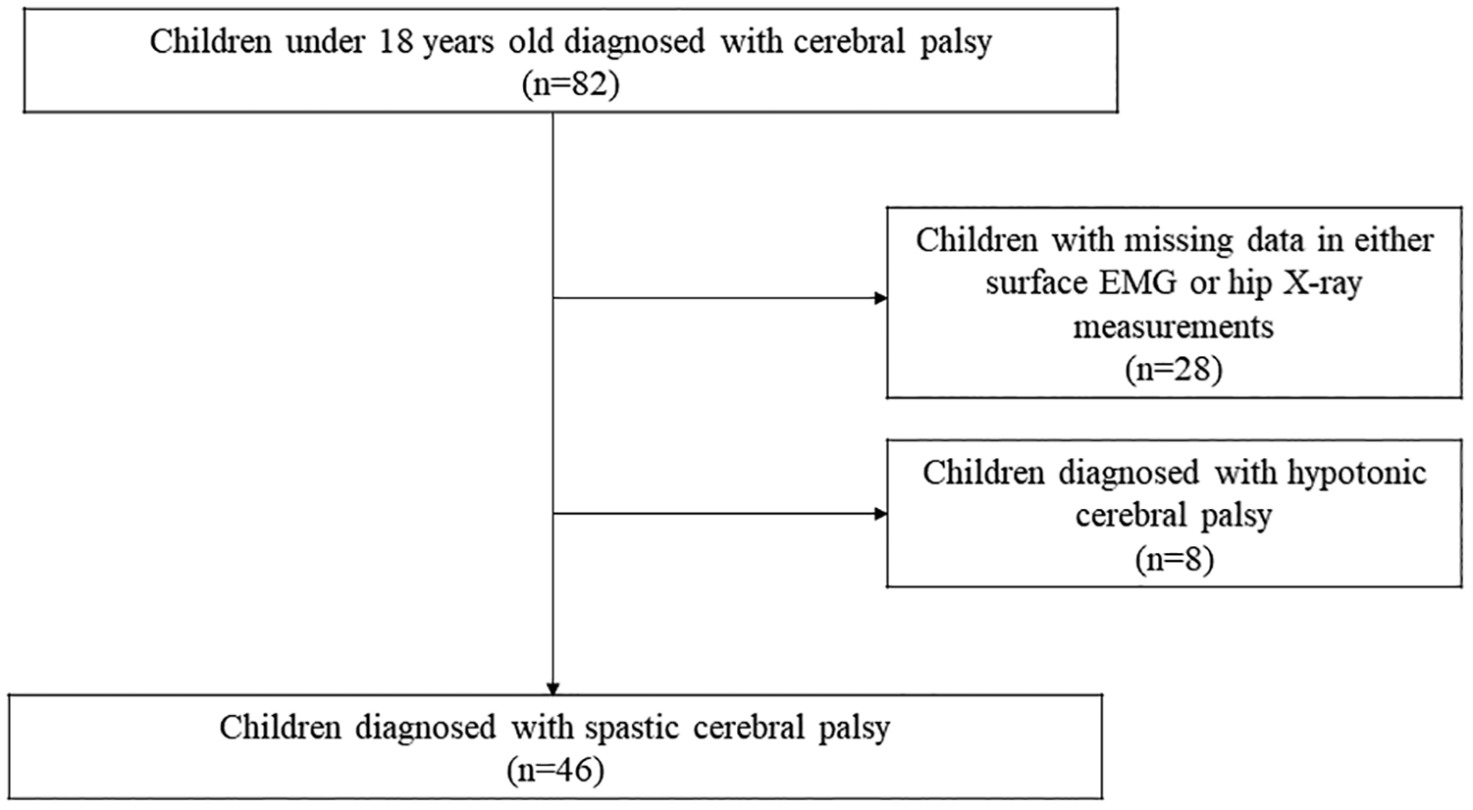
Flowchart of the study.

A total of 46 children with spastic CP were enrolled, comprising 32 males and 14 females (Fig. 2). Four participants had a history of orthopedic surgery, and 17 had previously received botulinum toxin injections. Notably, a substantial proportion of participants met high risk criteria for hip displacement, including MI ≥ 60% and annual MI progression ≥10%. Detailed demographic, spasticity, ROM, radiographic, and S-EMG characteristics are summarized in Tables 1 and 2.

**Table 1:** Demographic data.

*N* = 46		Average ± SD
Age (yr)		6.27 ± 3.00
Gender	Male	32
	Female	14
Operation		4
Botulinum toxin injection		17
Strength of the hip adductor muscles		16.02 ± 19.40
High risk	Operation	5 (10.86%)
	MI ≥ 60%	10 (21.74 %)
	Large MI for the age	6 (13.04%)
	Annual progression ≥ 10%	5 (10.86%)
MAS	Hip abductor with knee flexion	1.61 ± 1.19
	Hip abductor with knee extension	1.40 ± 1.17
	Hip flexor	1.54 ± 1.17
	Knee extensor	1.49 ± 1.48
ROM	Hip abduction with hip flexion at 90 degrees	46.73 ± 33.77
	Hip abduction with hip extension	31.33 ± 25.84
	knee flexion	112.78 ± 47.03
	knee extension	−35.22 ± 60.00
	hip IR	31.50 ± 16.69
	hip ER	44.10 ± 22.03

AI = acetabular index, ER = external rotation, IR = internal rotation, MAS = Modified Ashworth Scale, MI = migration index, PO = pelvic obliquity, ROM = range of motion, SD = standard deviation.

**Table 2:** Results of surface EMG and hip X-ray.

Factor		Average ± SD
Hip surface EMG when resting	Ch 1 (Lt. tensor fasciae latae)	7.48 ± 9.75
	Ch 2 (Rt. tensor fasciae latae)	13.21 ± 23.44
	Ch 3 (Lt. gluteus medius)	6.64 ± 6.94
	Ch 4 (Rt. gluteus medius)	6.23 ± 6.23
	Ch 5 (Lt. adductor longus)	19.31 ± 9.04
	Ch 6 (Rt. adductor longus)	12.29 ± 10.40
	Ch 7 (Lt. adductor magnus)	9.49 ± 8.05
	Ch 8 (Rt. adductor magnus)	11.12 ± 9.55
Hip surface EMG when spasticity	Ch 1 (Lt. tensor fasciae latae)	71.10 ± 67.99
	Ch 2 (Rt. tensor fasciae latae)	73.79 ± 108.13
	Ch 3 (Lt. gluteus medius)	33.06 ± 62.59
	Ch 4 (Rt. gluteus medius)	24.25 ± 29.86
	Ch 5 (Lt. adductor longus)	56.67 ± 46.13
	Ch 6 (Rt. adductor longus)	50.20 ± 46.75
	Ch 7 (Lt. adductor magnus)	56.14 ± 47.64
	Ch 8 (Rt. adductor magnus)	52.19 ± 47.10
Hip surface EMG when spasticity with hip abductor bar	Ch 1 (Lt. tensor fasciae latae)	42.61 ± 60.47
	Ch 2 (Rt. tensor fasciae latae)	47.96 ± 82.71
	Ch 3 (Lt. gluteus medius)	20.30 ± 49.61
	Ch 4 (Rt. gluteus medius)	19.43 ± 27.13
	Ch 5 (Lt. adductor longus)	35.98 ± 45.92
	Ch 6 (Rt. adductor longus)	35.65 ± 44.41
	Ch 7 (Lt. adductor magnus)	33.64 ± 43.45
	Ch 8 (Rt. adductor magnus)	30.13 ± 39.90
Spine surface EMG when spasticity	Ch 1 (Lt. rectus abdominis)	36.04 ± 38.36
	Ch 2 (Rt. rectus abdominis)	32.50 ± 40.25
	Ch 3 (Lt. external abdominal obliques)	34.24 ± 34.15
	Ch 4 (Rt. external abdominal obliques)	31.90 ± 29.17
	Ch 5 (Lt. L3 erector spinae)	24.66 ± 42.15
	Ch 6 (Rt. L3 erector spinae)	27.81 ± 49.99
	Ch 7 (Lt. L3 multifidus)	18.24 ± 25.62
	Ch 8 (Rt. L3 multifidus)	21.06 ± 29.73
Hip X-ray	MI	51.31 ± 26.71
	Coxa valga	158.57 ± 8.82
	AI	26.40 ± 8.17
	PO	3.62 ± 2.59

AI = acetabular index, Ch = channel, EMG = electromyography, Lt = left, MI = migration index, PO = pelvic obliquity, Rt = right, SD = standard deviation.

### 3.2. Multiple regression analysis of MI and physical measurements, including S-EMG

**Figure 3.**
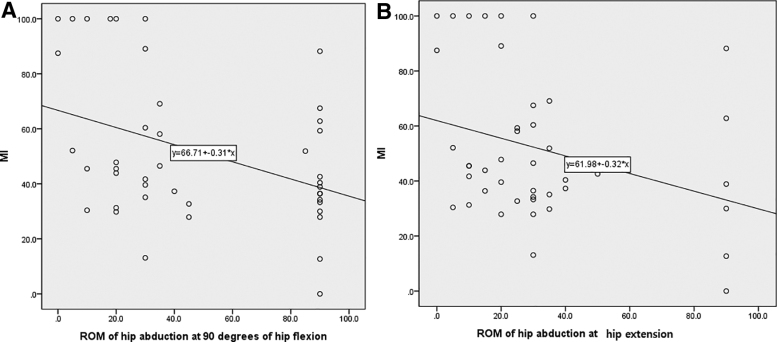
Scatter plot indicating a significant correlation between MI and the ROM of hip abduction with a hip flexion of (A) 90 degrees and (B) 0 degrees.

MI showed significant moderate negative correlations with the ROM of hip abduction at both 90-degree hip flexion (Table 3, Figure 3A) and 0-degree hip flexion (Table 3, Figure 3B).

**Table 3:** Correlation between ROM and hip deformity parameters (with 95% CI).

Factors	MI			Coxa valga			AI			PO		
	*r*	95% CI	*P*	*r*	95% CI	*P*	*r*	95% CI	*P*	*r*	95% CI	*P*
ROM of Hip abduction with hip flexion 90°	−0.40	(−0.62 to −0.11)	**0.01** *	−0.04	(−0.34 to 0.27)	0.80	−0.17	(−0.45 to 0.14)	0.29	−0.12	(−0.41 to 0.20)	0.47
ROM of Hip abduction with hip flexion 0°	−0.31	(−0.56 to −0.02)	**0.04** *	0.04	(−0.27 to 0.34)	0.82	−0.17	(−0.45 to 0.14)	0.28	−0.04	(−0.34 to 0.28)	0.83
ROM of knee flexion	−0.22	(−0.48 to 0.09)	0.17	−0.17	(−0.45 to 0.14)	0.29	0.02	(−0.28 to 0.33)	0.88	−0.10	(−0.40 to 0.22)	0.53
ROM of knee extension	−0.17	(−0.44 to 0.14)	0.29	−0.05	(−0.35 to 0.26)	0.75	−0.17	(−0.45 to 0.14)	0.29	−0.20	(−0.48 to 0.12)	0.22
ROM of hip internal rotation	−0.20	(−0.48 to 0.12)	0.22	−0.19	(−0.48 to 0.13)	0.24	−0.07	(−0.37 to 0.25)	0.69	0.04	(−0.28 to 0.35)	0.82
ROM of hip external rotation	−0.08	(−0.39 to 0.24)	0.63	−0.10	(−0.40 to 0.23)	0.56	−0.12	(−0.42 to 0.20)	0.46	0.15	(−0.18 to 0.45)	0.37

AI = acetabular index, CI = confidence interval, MI = migration index, PO = pelvic obliquity, ROM = range of motion.

* *P* <.05.

Multiple regression analysis revealed that MI has a negative correlation with the ROM of hip abduction with a hip flexion of 90 degrees, and the relationship equation is as follows:


$$\begin{array}{c} \begin{array}{cc} & MI=60.51-0.26\\ & \times \left(ROM\;of\;hip\;abduction\;with\;hip\;flexion\;of\;90\;degrees\right) \end{array} \end{array}$$

Stepwise multiple regression analysis identified ROM of hip abduction at 90-degree hip flexion as the only significant independent predictor of MI (*β* = −0.312, 95% CI: −0.539 to −0.084, standardized *β* = −0.397, *R*^2^ = 0.157, *P* = .008). Residual analysis confirmed the assumptions of linearity, normality, and homoscedasticity. No multicollinearity was present as a single predictor was retained (variance inflation factor=1.0). The correlation between MI and ROM of hip abduction at 90-degree and 0-degree hip flexion is presented with 95% CIs in Table 3.

### 3.3. Correlation analysis of Femur neck-shaft angle and physical measurements, including S-EMG

The femoral neck-shaft angle showed a significant moderate negative correlation with the Abductor Sum/Adductor Sum ratio during spasticity, suggesting that relatively greater abductor activation is associated with a reduced femoral neck-shaft angle and a lower likelihood of coxa valga (Table 4). Regression analysis further confirmed this association (*β* = −6.453, 95% CI: −11.990–−0.916, standardized *β* = −0.349, *R*^2^ = 0.122, *P* = .023). Residual analysis confirmed the assumptions of linearity, normality, and homoscedasticity. No multicollinearity was present as a single predictor was retained (variance inflation factor=1.0).

**Table 4:** Correlation between surface EMG parameters and hip deformity parameters (with 95% CI).

Factor		MI			Coxa valga			AI			PO		
		*r*	95% CI	*P*	*r*	95% CI	*P*	*r*	95% CI	*P*	*r*	95% CI	*P*
**Hip surface EMG when spasticity**	Ch 1 (Lt. TFL)	−0.01	(−0.31 to 0.29)	.96	−0.14	(−0.42 to 0.18)	.39	0.09	(−0.22 to 0.39)	.56	−0.23	(−0.50 to 0.09)	.16
	Ch 2 (Rt. TFL)	−0.00	(−0.30 to 0.30)	.98	−0.26	(−0.52 to 0.05)	.10	−0.06	(−0.36 to 0.25)	.69	0.03	(−0.28 to 0.34)	.84
	Ch 3 (Lt. glut. med.)	0.13	(−0.18 to 0.41)	.42	−0.05	(−0.35 to 0.25)	.73	0.32	(0.02–0.57)	**.04** *	−0.13	(−0.42 to 0.19)	.43
	Ch 4 (Rt. glut. med.)	−0.07	(−0.36 to 0.24)	.66	−0.14	(−0.42 to 0.17)	.39	−0.09	(−0.39 to 0.22)	.56	−0.15	(−0.44 to 0.17)	.36
	Ch 5 (Lt. add. long.)	−0.12	(−0.41 to 0.18)	.44	−0.10	(−0.39 to 0.21)	.52	−0.10	(−0.39 to 0.21)	.52	−0.19	(−0.47 to 0.13)	.25
	Ch 6 (Rt. add. long.)	−0.06	(−0.35 to 0.25)	.72	−0.00	(−0.30 to 0.30)	1.00	0.01	(−0.29 to 0.31)	.94	−0.21	(−0.49 to 0.11)	.20
	Ch 7 (Lt. add. mag.)	−0.09	(−0.38 to 0.22)	.58	0.08	(−0.23 to 0.37)	.63	0.04	(−0.27 to 0.34)	.82	−0.24	(−0.51 to 0.08)	.13
	Ch 8 (Rt. add. mag.)	−0.13	(−0.41 to 0.18)	.41	−0.15	(−0.43 to 0.17)	0.36	−0.04	(−0.34 to 0.27)	.82	−0.16	(−0.45 to 0.16)	.32
**Hip surface EMG when spasticity with abductor bar**	Ch 1 (Lt. TFL)	0.11	(−0.19 to 0.40)	.47	−0.13	(−0.41 to 0.18)	.42	0.13	(−0.18 to 0.42)	.42	−0.08	(−0.38 to 0.24)	.63
	Ch 2 (Rt. TFL)	0.03	(−0.27 to 0.33)	.84	−0.23	(−0.50 to 0.08)	.15	−0.08	(−0.38 to 0.23)	.61	0.02	(−0.29 to 0.33)	.90
	Ch 3 (Lt. glut. med.)	0.13	(−0.17 to 0.42)	.40	−0.07	(−0.36 to 0.24)	.67	0.31	(0.00–0.56)	**.05** *	−0.07	(−0.38 to 0.24)	.65
	Ch 4 (Rt. glut. med.)	0.22	(−0.09 to 0.49)	.16	−0.00	(−0.31 to 0.30)	.98	0.06	(−0.25 to 0.36)	.70	−0.02	(−0.33 to 0.30)	.92
	Ch 5 (Lt. add. long.)	0.07	(−0.23 to 0.37)	.64	−0.12	(−0.41 to 0.19)	.46	0.07	(−0.24 to 0.37)	.66	0.04	(−0.27 to 0.35)	.80
	Ch 6 (Rt. add. long.)	−0.01	(−0.31 to 0.29)	.97	−0.12	(−0.41 to 0.19)	.46	−0.10	(−0.40 to 0.21)	.51	−0.09	(−0.39 to 0.23)	.59
	Ch 7 (Lt. add. mag.)	0.07	(−0.24 to 0.36)	.66	−0.02	(−0.32 to 0.29)	.90	0.04	(−0.27 to 0.34)	.81	0.02	(−0.29 to 0.33)	.91
	Ch 8 (Rt. add. mag.)	0.00	(-0.30 to 0.30)	.99	−0.14	(−0.42 to 0.18)	.39	−0.03	(−0.33 to 0.27)	.83	−0.04	(−0.35 to 0.27)	.80
**Spine Surface EMG when spasticity**	Ch 1 (Lt. rect. abd.)	−0.06	(−0.35 to 0.24)	.70	−0.14	(−0.42 to 0.17)	.39	0.06	(−0.25 to 0.36)	.70	−0.03	(−0.34 to 0.29)	.86
	Ch 2 (Rt. rect. abd.)	−0.03	(−0.33 to 0.27)	.85	0.08	(−0.23 to 0.37)	.63	0.02	(−0.29 to 0.32)	.92	−0.05	(−0.36 to 0.26)	.75
	Ch 3 (Lt. ext. obl.)	0.13	(−0.17 to 0.42)	.39	−0.14	(−0.42 to 0.17)	.38	0.37	(0.08–0.61)	**.02** *	−0.03	(−0.34 to 0.28)	.86
	Ch 4 (Rt. ext. obl.)	0.09	(−0.21 to 0.38)	.55	−0.12	(−0.41 to 0.19)	.46	0.12	(−0.19 to 0.41)	.43	0.05	(−0.27 to 0.35)	.78
	Ch 5 (Lt. L3 erect.)	−0.04	(−0.34 to 0.26)	.80	−0.14	(−0.43 to 0.17)	.37	0.17	(−0.14 to 0.45)	.27	−0.20	(−0.49 to 0.11)	.20
	Ch 6 (Rt. L3 erect.)	0.17	(−0.14 to 0.44)	.29	−0.02	(−0.32 to 0.29)	.90	0.45	(0.16–0.66)	**.00** *	−0.17	(−0.46 to 0.15)	.30
	Ch 7 (Lt. L3 multif.)	0.18	(−0.12 to 0.46)	.24	0.05	(−0.26 to 0.35)	.76	0.23	(−0.08 to 0.50)	.15	0.11	(−0.21 to 0.40)	.52
	Ch 8 (Rt. L3 multif.)	0.16	(−0.15 to 0.44)	.32	−0.04	(−0.34 to 0.26)	.78	0.24	(−0.07 to 0.51)	.12	−0.24	(−0.51 to 0.08)	.14

add. long. = adductor longus, add. mag. = adductor magnus, AI = acetabular index, CI = confidence interval, EMG = electromyography, erect. = erector spinae, ext. obl. = external abdominal oblique, glut. med. = gluteus medius, Lt = left, MI = migration index, multif. = multifidus, PO = pelvic obliquity, rect. abd. = rectus abdominis, Rt = right, SD = standard deviation, TFL = tensor fasciae latae.

* *P* < .05.

### 3.4. Correlation analysis of AI and physical measurements, including S-EMG

We analyzed the relationship between AI and S-EMG. AI showed a significant moderate positive correlation with the left gluteus medius, left external abdominal oblique, and right L3 erector spinae (Table 4).

### 3.5. Correlation analysis of PO and physical measurements, including S-EMG

No significant correlations were identified between PO and any of the physical measurements or S-EMG parameters (Table 4).

## 4. Discussion

This was an exploratory, hypothesis-generating study examining the associations between hip deformity parameters and physical measurements, including ROM and S-EMG, in nonambulatory children with spastic CP. As no formal a priori power calculation was performed, the findings should be interpreted with caution and considered as preliminary evidence to inform the design of future confirmatory studies with adequate sample sizes. The results demonstrated that MI was significantly related to the ROM of hip abduction, and the femoral neck-shaft angle was significantly related to the abductor sum/adductor sum ratio. This suggests that hip deformities, such as hip subluxation and coxa valga, are associated with hip abduction ROM and the balance between hip abductor strength and hip adductor strength.

According to previous studies, the hip abduction does not correlate with MI.^[23,24]^ However, in this study, the ROM of hip abduction with 90 degrees of hip flexion and with 0 degrees of hip flexion showed a significant moderate negative correlation. Additionally, multiple regression analysis revealed that MI was negatively correlated with the ROM of hip abduction with 90 degrees of hip flexion. Prior research on hip geometry in children with CP has highlighted significant differences compared to typically developing children. In patients with spastic CP, muscle imbalance generates abnormal forces in the hip joint.^[4,25]^ Over time, excessive activation of the adductors, hip flexors, and hamstrings disrupts the normal joint reactive forces, progressively leading to proximal femoral subluxation from the acetabulum.^[23,24]^ This muscle imbalance not only affects hip abduction but also leads to difficulty in hip extension. In this study, Pearson’s correlation analysis showed associations between hip abduction ROM in 90° flexion and hip extension with MI; however, multiple regression identified only abduction ROM in 90° flexion as a significant negative predictor. This implies that restricted ROM of the tensor fasciae latae and psoas muscles may play a more critical role than the gluteus maximus in hip subluxation, highlighting the importance of targeting these muscles in preventive strategies.

Coxa valga is a known risk factor for hip displacement in patients with CP.^[26]^ Previous studies have shown that children with CP and higher head shaft angles have a 1.6-fold higher risk of hip displacement than children with CP and the same GMFCS level, age, and hip MI.^[27]^ Even in cases of hip reconstruction surgery in children with CP, patients with a higher preoperative neck-shaft angle showed significantly increased failure rates, resulting in revision surgery. Therefore, interventions to prevent coxa valga are important to prevent hip displacement and further redislocation in patients with CP.^[28]^ A prior study indicated that hip dislocation in children with CP primarily occurs owing to an imbalance in muscle strength. This type of hip deformity occurs when the adductive force surpasses the abductive force during spasticity, leading to the femoral head shifting outward from the acetabulum.^[29]^ This study demonstrated that when the abductor sum is greater than the ratio of the abductor sum to the adductor sum, the increase in the femur shaft-neck angle is suppressed, preventing coxa valga. This study suggests that the abductor sum should be greater than the adductor sum to reduce the risk of coxa valga. Therefore, rehabilitation for nonambulatory children with CP may benefit from focusing not only on ROM exercises for hip abduction but also on strengthening the hip abductor muscles, although interventional studies are needed to confirm these associations. Based on the observed associations, neuromuscular electrical stimulation targeting hip abductors represents a potentially interesting therapeutic avenue; however, its efficacy in preventing hip dislocation in this population has not been established and requires validation through future controlled interventional studies. Additionally, abductor muscle strengthening and adductor-targeting interventions such as botulinum toxin injection are theoretically plausible strategies based on the observed muscle imbalance findings; however, these approaches should be investigated in prospective controlled studies before clinical recommendations can be made.

Several hip surveillance programs recommend regular radiographic hip evaluations.^[30]^ For children with CP classified as GMFCS IV–V, the Scandinavian model recommends annual radiographic evaluations until the age of 8 years, whereas the Australian model recommends radiographic evaluations every 6 to 12 months until the age of 7 years.^[31,32]^ A previous study showed that exposure to diagnostic radiation below the waist may increase the incidence of testicular germ cell tumors.^[33]^ Sensitivity to radiation-induced cancer is up to ten times higher in children than in adults. Until approximately the age of 10, the risk of cancer development reaches 15% per sievert (Sv), whereas in adulthood, the risk decreases to 1% to 2% per Sv.^[34]^ Therefore, the risk of radiation-induced cancer is significantly higher in children, particularly those aged <10 years.^[34]^ For the same examination, the effective dose was higher in children than in adults.^[35]^ Additionally, because of the closer anatomical proximity of organs in children, adjacent organs may receive higher radiation exposure when imaging the target region. Consequently, the risk of radiation exposure to the surrounding organs is greater in pediatric patients. Hip radiography is essential for hip surveillance in nonambulatory children with CP. This study proposes an alternative approach to minimize the risk of cancer associated with radiation exposure. By initially assessing the ROM of hip abduction and utilizing S-EMG, both of which do not involve radiation exposure, groups at high risk for hip subluxation could be identified. Limiting hip radiography to high risk cases may help reduce unnecessary radiation exposure.

Acetabular dysplasia refers to an underdeveloped acetabulum characterized by structural deficiencies in shape and orientation, leading to inadequate coverage of the femoral head.^[36–38]^ AI is an objective measure of acetabular dysplasia that can be used to determine its severity. This insufficient coverage can result in excessive mechanical stress on the acetabular articular cartilage and labrum, potentially accelerating degenerative changes. A previous study indicated that acetabular dysplasia increases in proportion to both MI and GMFCS levels.^[39]^ In cases of severe acetabular dysplasia, Dega osteotomy is the most widely recognized surgical procedure for managing acetabular dysplasia in children with CP.^[40]^ In this study, AI was positively correlated with the RMS of S-EMG signals in the left gluteus medius, left external abdominal oblique, and right L3 erector spinae muscles. This finding may be attributed to the fact that an increase in AI can lead to acetabular dysplasia, which may subsequently progress to hip osteoarthritis in adults. According to previous studies on end-stage hip osteoarthritis in adults, the increased mean muscle amplitudes of the gluteus medius may suggest that the hip abductor muscles recruit a greater number of motor units or engage the available motor units more extensively than the matched controls to accomplish the same functional task.^[41,42]^ Although the present study focused on nonambulatory children with CP, this compensatory mechanism for muscle weakness associated with hip deformity in adults may help explain the observed results.

This study has several notable strengths. First, data were collected across 3 rehabilitation units, enhancing the external validity and generalizability of the findings beyond a single-institution setting. Second, objective and quantitative measurement tools were employed throughout, including standardized radiographic parameters (MI, femoral neck-shaft angle, AI, and PO) and wireless S-EMG analysis, thereby minimizing reliance on subjective clinical assessment. To reduce potential sources of measurement bias, all ROM and spasticity assessments were performed by trained physiatrists using standardized protocols, and S-EMG recordings were obtained twice, with mean values used for analysis, reducing the influence of random measurement error. Furthermore, the Net Adduction Index incorporated moment-arm length weighting for each muscle, providing a more biomechanically accurate representation of the adductor-abductor force balance than simple RMS amplitude comparisons alone. Finally, this study proposes a clinically meaningful strategy for early risk identification using non-radiation-based tools such as ROM measurement and S-EMG, which may help reduce unnecessary radiation exposure in this vulnerable pediatric population while maintaining the effectiveness of hip surveillance.

### 4.1. Limitation

This study had several limitations. First, the sample size was relatively small (*n* = 46), reflecting the strict inclusion criteria targeting a narrowly defined and clinically severe subpopulation: children with spastic CP classified as GMFCS levels IV–V aged 2 to 10 years, which limits generalizability to older children or adults. A formal a priori power calculation was not conducted; therefore, all findings should be regarded as hypothesis-generating rather than confirmatory, and future studies with larger sample sizes and prospectively determined power calculations are warranted.

Second, the absence of a typically developing control group limits the interpretability of S-EMG and ROM findings, as normative reference values were unavailable. Additionally, S-EMG data were not normalized to the percentage of maximum voluntary contraction, as this was not feasible in this severely affected population, limiting cross-study comparability. Future studies should incorporate age-matched controls and explore appropriate normalization methods such as submaximal reference contractions.

Third, formal inter-rater and intra-rater reliability data for ROM, MAS, manual muscle strength testing, and electrode placement were not collected, and regression models did not adjust for potential confounders including age, GMFCS level, botulinum toxin injection history, and prior orthopedic surgery, due to the small sample size. Multiple correlation analyses were conducted without correction for multiple comparisons, increasing the risk of Type I error; strict corrections were intentionally avoided to preserve sensitivity in this exploratory study.

Finally, this cross-sectional design precludes causal inference, and the findings should be interpreted as associative rather than confirmatory.

## 5. Conclusion

This study suggests that in nonambulatory children with spastic CP, hip deformities are closely associated with hip abduction ROM and the abductor-to-adductor muscle strength ratio. A higher abductor-to-adductor ratio was associated with reduced femoral neck-shaft angle, suggesting a potential protective role against coxa valga and hip displacement. Future interventional studies are needed to confirm whether rehabilitation focused on hip abductor strengthening and ROM exercises can prevent hip deformity progression. Non-radiation-based tools like ROM and S-EMG assessments can aid in early detection while minimizing radiation exposure. Further large-scale, long-term studies are needed to confirm these findings and optimize preventive strategies.

## Author contributions

**Conceptualization:** Ju Seok Ryu, Yulhyun Park, Eunseo Choi, Jee Hyun Suh.

**Data curation:** Ju Seok Ryu, Yulhyun Park, Joonyoung Jang, Hyun Jin Kim, Eunseo Choi, Jaewon Lee.

**Formal analysis:** Ju Seok Ryu, Yulhyun Park, Joonyoung Jang, Hyun Jin Kim, Eunseo Choi.

**Supervision:** Ju Seok Ryu.

**Methodology:** Joonyoung Jang, Jee Hyun Suh.

**Resources:** Hyun Jin Kim.

**Project administration:** Jee Hyun Suh.

**Validation:** Jee Hyun Suh.

**Writing – original draft:** Ju Seok Ryu, Jee Hyun Suh.

**Writing – review & editing:** Jee Hyun Suh.



## References

[1] Rosenbloom L. Definition and classification of cerebral palsy. Definition, classification, and the clinician. Dev Med Child Neurol Suppl. 2007;109:43. 17370483 10.1111/j.1469-8749.2007.tb12629.x

[2] Hagberg B Hagberg G Olow I von Wendt L. The changing panorama of cerebral palsy in Sweden. V. The birth year period 1979-82. Acta Paediatr Scand. 1989;78:283–90. 2784617 10.1111/j.1651-2227.1989.tb11071.x

[3] Hagglund G Andersson S Duppe H Lauge-Pedersen H Nordmark E Westbom L. Prevention of dislocation of the hip in children with cerebral palsy. The first ten years of a population-based prevention programme. J Bone Joint Surg Br. 2005;87:95–101. 15686244

[4] Hagglund G Lauge-Pedersen H Wagner P. Characteristics of children with hip displacement in cerebral palsy. BMC Musculoskelet Disord. 2007;8:101. 17963501 10.1186/1471-2474-8-101 PMC2194677

[5] Terjesen T. The natural history of hip development in cerebral palsy. Dev Med Child Neurol. 2012;54:951–7. 22881288 10.1111/j.1469-8749.2012.04385.x

[6] Adjenti SK Louw G Jelsma J Unger M. An electromyographic study of abdominal muscle activity in children with spastic cerebral palsy. S Afr J Physiother. 2017;73:341. 30135898 10.4102/sajp.v73i1.341 PMC6093119

[7] Giuca G Sanzarello I Marletta DA Calaciura S Nanni M Leonetti D. Incidence and risk factors of hip dislocation in children with cerebral palsy: a systematic review and pooled analysis. J Clin Orthop Trauma. 2025;69:103141. 40814403 10.1016/j.jcot.2025.103141 PMC12345335

[8] Huser A Mo M Hosseinzadeh P. Hip surveillance in children with cerebral palsy. Orthop Clin North Am. 2018;49:181–90. 29499819 10.1016/j.ocl.2017.11.006

[9] Malone A Tanner G French HP. Longitudinal relationship between hip displacement and hip function in children and adolescents with cerebral palsy: a scoping review. Dev Med Child Neurol. 2025;67:450–62. 39572923 10.1111/dmcn.16175 PMC11875528

[10] Ryu JS Suh JH. Optimal frequency of physical therapy in young children with cerebral palsy: a retrospective pilot study. Dev Neurorehabil. 2023;26:37–43. 36384414 10.1080/17518423.2022.2147595

[11] Almeida da Silva LC Hori Y Kaymaz B. Femoral neck-shaft angle changes based on the severity of neurologic impairment in children with cerebral palsy and spinal muscular atrophy. J Child Orthop. 2024;18:523–30. 39493870 10.1177/18632521241277023 PMC11528760

[12] von Elm E Altman DG Egger M Pocock SJ Gotzsche PC Vandenbroucke JP; STROBE Initiative. The Strengthening the Reporting of Observational Studies in Epidemiology (STROBE) statement: guidelines for reporting observational studies. Lancet. 2007;370:1453–7. 18064739 10.1016/S0140-6736(07)61602-X

[13] Palisano RJ Rosenbaum P Bartlett D Livingston MH. Content validity of the expanded and revised gross motor function classification system. Dev Med Child Neurol. 2008;50:744–50. 18834387 10.1111/j.1469-8749.2008.03089.x

[14] Reimers J. The stability of the hip in children. A radiological study of the results of muscle surgery in cerebral palsy. Acta Orthop Scand Suppl. 1980;184:1–100. 6930145 10.3109/ort.1980.51.suppl-184.01

[15] Chung CY Lee KM Park MS Lee SH Choi IH Cho TJ. Validity and reliability of measuring femoral anteversion and neck-shaft angle in patients with cerebral palsy. J Bone Joint Surg Am. 2010;92:1195–205. 20439666 10.2106/JBJS.I.00688

[16] Tonnis D. Normal values of the hip joint for the evaluation of X-rays in children and adults. Clin Orthop Relat Res. 1976;119:39–47. 954321

[17] Heidt C Hollander K Wawrzuta J. The radiological assessment of pelvic obliquity in cerebral palsy and the impact on hip development. Bone Joint J. 2015;97-B:1435–40. 26430022 10.1302/0301-620X.97B10.35390

[18] Ansari NN Naghdi S Arab TK Jalaie S. The interrater and intrarater reliability of the Modified Ashworth Scale in the assessment of muscle spasticity: limb and muscle group effect. NeuroRehabilitation. 2008;23:231–7. 18560139

[19] Rainoldi A Melchiorri G Caruso I. A method for positioning electrodes during surface EMG recordings in lower limb muscles. J Neurosci Methods. 2004;134:37–43. 15102501 10.1016/j.jneumeth.2003.10.014

[20] Kim S Lee D Ko JY. The mechanism of hip dislocation related to the use of abduction bar and hip compression bandage in patients with spastic cerebral palsy. Am J Phys Med Rehabil. 2019;98:1125–32. 31268886 10.1097/PHM.0000000000001261

[21] Neumann DA. Kinesiology of the hip: a focus on muscular actions. J Orthop Sports Phys Ther. 2010;40:82–94. 20118525 10.2519/jospt.2010.3025

[22] Dancey CP Reidy J. Statistics without Maths for Psychology. Pearson Prentice Hall; 2007.

[23] Dobson F Boyd RN Parrott J Nattrass GR Graham HK. Hip surveillance in children with cerebral palsy. Impact on the surgical management of spastic hip disease. J Bone Joint Surg Br. 2002;84:720–6. 12188492 10.1302/0301-620x.84b5.12398

[24] Wynter M Gibson N Willoughby KL; National Hip Surveillance Working Group. Australian hip surveillance guidelines for children with cerebral palsy: 5-year review. Dev Med Child Neurol. 2015;57:808–20. 25846730 10.1111/dmcn.12754

[25] Soo B Howard JJ Boyd RN. Hip displacement in cerebral palsy. J Bone Joint Surg Am. 2006;88:121–9. 16391257 10.2106/JBJS.E.00071

[26] Lee KM Kang JY Chung CY. Clinical relevance of valgus deformity of proximal femur in cerebral palsy. J Pediatr Orthop. 2010;30:720–5. 20864860 10.1097/BPO.0b013e3181edba2a

[27] Hermanson M Hagglund G Riad J Wagner P. Head-shaft angle is a risk factor for hip displacement in children with cerebral palsy. Acta Orthop. 2015;86:229–32. 25428756 10.3109/17453674.2014.991628 PMC4404776

[28] Minaie A Gordon JE Schoenecker P Hosseinzadeh P. Failure of hip reconstruction in children with cerebral palsy: what are the risk factors? J Pediatr Orthop. 2022;42:e78–82. 34657091 10.1097/BPO.0000000000001989

[29] Miller F Slomczykowski M Cope R Lipton GE. Computer modeling of the pathomechanics of spastic hip dislocation in children. J Pediatr Orthop. 1999;19:486–92. 10412998 10.1097/00004694-199907000-00012

[30] Shrader MW Wimberly L Thompson R. Hip surveillance in children with cerebral palsy. J Am Acad Orthop Surg. 2019;27:760–8. 30998565 10.5435/JAAOS-D-18-00184

[31] Robb JE Hagglund G. Hip surveillance and management of the displaced hip in cerebral palsy. J Child Orthop. 2013;7:407–13. 24432103 10.1007/s11832-013-0515-6 PMC3838516

[32] Wynter M Gibson N Kentish M Love S Thomason P Kerr Graham H. The development of Australian standards of care for hip surveillance in children with cerebral palsy: how did we reach consensus? J Pediatr Rehabil Med. 2011;4:171–82. 22207094 10.3233/PRM-2011-0173

[33] Nead KT Mitra N Weathers B. Lower abdominal and pelvic radiation and testicular germ cell tumor risk. PLoS One. 2020;15:e0239321. 33175879 10.1371/journal.pone.0239321 PMC7657535

[34] Hall EJ. Lessons we have learned from our children: cancer risks from diagnostic radiology. Pediatr Radiol. 2002;32:700–6. 12244457 10.1007/s00247-002-0774-8

[35] Ware DE Huda W Mergo PJ Litwiller AL. Radiation effective doses to patients undergoing abdominal CT examinations. Radiology. 1999;210:645–50. 10207462 10.1148/radiology.210.3.r99mr05645

[36] Klaue K Durnin CW Ganz R. The acetabular rim syndrome. A clinical presentation of dysplasia of the hip. J Bone Joint Surg Br. 1991;73:423–9. 1670443 10.1302/0301-620X.73B3.1670443

[37] Gala L Clohisy JC Beaule PE. Hip dysplasia in the young adult. J Bone Joint Surg Am. 2016;98:63–73. 26738905 10.2106/JBJS.O.00109

[38] Disantis AE Martin RL Enseki K Spaid V McClincy M. Non-operative rehabilitation principles for use in individuals with acetabular dysplasia: a North American-based Delphi study. Int J Sports Phys Ther. 2023;18:1331–45. 38050551 10.26603/001c.89265 PMC10693488

[39] Chung MK Zulkarnain A Lee JB. Functional status and amount of hip displacement independently affect acetabular dysplasia in cerebral palsy. Dev Med Child Neurol. 2017;59:743–9. 28432692 10.1111/dmcn.13437

[40] Chung CY Choi IH Cho TJ Yoo WJ Lee SH Park MS. Morphometric changes in the acetabulum after Dega osteotomy in patients with cerebral palsy. J Bone Joint Surg Br. 2008;90:88–91. 18160506 10.1302/0301-620X.90B1.19674

[41] Dwyer MK Stafford K Mattacola CG Uhl TL Giordani M. Comparison of gluteus medius muscle activity during functional tasks in individuals with and without osteoarthritis of the hip joint. Clin Biomech (Bristol). 2013;28:757–61. 23911109 10.1016/j.clinbiomech.2013.07.007

[42] Ling SM Conwit RA Talbot L. Electromyographic patterns suggest changes in motor unit physiology associated with early osteoarthritis of the knee. Osteoarthritis Cartilage. 2007;15:1134–40. 17543548 10.1016/j.joca.2007.03.024 PMC2259251

