# Supplementary material for: Factors associated with hip deformity in children with nonambulatory spastic cerebral palsy
Source: Medicine (Baltimore). 2026 Jun 26;105(26):e49553. doi: 10.1097/MD.0000000000049553 (PMC13313724; doi:10.1097/MD.0000000000049553)
Supplement: Supplementary file 1 [file medi-105-e49553-s001.docx]

Supplemental Table 1. Attachment site of surface EMG electrodes

| Area | Channel | Muscle | Anatomical Surface Location |
| --- | --- | --- | --- |
| Lower extremity muscle | 1,2 | Adductor longus m.  (Lt: 1, Rt:2) | Attached on the 0/4-1/4 point of the line connecting pubic symphysis and the midpoint of medial collateral ligament |
|  | 3,4 | Adductor magus m.  (Lt: 3, Rt:4) | Attached on the 3/4-4/4 point of the line connecting pubic symphysis and the midpoint of medial collateral ligament |
|  | 5,6 | Tensor fascia lata m.  (Lt: 5, Rt:6) | Attached on the 1/8-3/8 point of the line connecting ASIS and the midpoint of lateral collateral ligament |
|  | 7,8 | Gluteus medius m.  (Lt: 7, Rt:8) | Attached on the superior-lateral quadrant of hip |
| Core muscle | 1,2 | Rectus abdominis m.  (Lt: 1, Rt:2) | Attached on the 3 cm above the umbilicus and 3 cm away from the median plane |
|  | 3,4 | External oblique m.  (Lt:3, Rt:4) | Attached on the midway between ASIS and the lowest point on the subcostal angle in the mid-axillary line always |
|  | 5,6 | L3 erector spinae m.  (Lt: 5, Rt:6) | Attached on the |
|  | 7,8 | L3 multifidus m.  (Lt: 7, Rt:8) | Attached on the |

ASIS, anterior superior iliac spine; Lt: left; Rt, right
